# A Data-Driven Optimized Mechanism for Improving Online Collaborative Learning: Taking Cognitive Load into Account

**DOI:** 10.3390/ijerph19126984

**Published:** 2022-06-07

**Authors:** Linjie Zhang, Xizhe Wang, Tao He, Zhongmei Han

**Affiliations:** 1 Key Laboratory of Intelligent Education Technology and Application of Zhejiang Province, Zhejiang Normal University, Jinhua 321004, China; ljzhang@zjnu.edu.cn (L.Z.); zm.han@zjnu.edu.cn (Z.H.); 2 School of Information Technology in Education, South China Normal University, Guangzhou 510631, China; tao.he2016@gmail.com

**Keywords:** online collaborative learning, cognitive load, optimized mechanism, system dynamics modeling

## Abstract

Research on online collaborative learning has explored various methods of collaborative improvement. Recently, learning analytics have been increasingly adopted for ascertaining learners’ states and promoting collaborative performance. However, little effort has been made to investigate the transformation of collaborative states or to consider cognitive load as an essential factor for collaborative intervention. By bridging collaborative cognitive load theory and system dynamics modeling methods, this paper revealed the transformation of online learners’ collaborative states through data analysis, and then proposed an optimized mechanism to ameliorate online collaboration. A quasi-experiment was conducted with 91 college students to examine the potential of the optimized mechanism in collaborative state transformation, awareness of collaboration, learning achievement, and cognitive load. The promising results demonstrated that students learning with the optimized mechanism performed significantly differently in collaboration and knowledge acquisition, and no additional burden in cognitive load was noted.

## 1. Introduction

Online collaborative learning refers to the computer-mediated version of traditional face-to-face collaboration [1], which undertakes most activities of online knowledge co-construction, collaborative competencies growth, and emotional communication. The continuous advancements of technology-enhanced online environments have shown considerable strength in supporting richer interactions and better collaborative engagement [2,3,4]. However, it also encounters an increased number of challenges. Compared with traditional collaboration, the virtual and changeful online environment obstructs contiguous interaction [5,6]. Additionally, a lack of direct guidance and communication increases the complexity of online collaboration. All of these factors cause a high level of mental effort and mental load, resulting in cognitive overload and reducing the awareness of collaboration [3,7,8,9], which has been found to hinder collaboration ability development, learning engagement, and individual performance [8,10,11].

Regarding cognitive overload in collaborative learning, Kirschner et al. [5] proposed collaborative cognitive load theory (CCLT), providing insights into preventing an individual’s cognitive overload by designing general guidelines. As such, it revealed a sound theoretical basis for online collaborative learning design in the present study to reduce extra cognitive load and enhance online collaborative learning correspondingly. There has been some research devoted to improving collaboration through CCLT [12,13], while few studies have dealt with learners’ extra cognitive load aroused by intervention. In recent years, the power of digital technologies has been harnessed for online collaborative analysis and facilitation [14,15,16]. Moreover, a substantial investment has been made to ascertain online learners’ states, and then develop personalized interventions or support [17,18]. However, little is known about how learners’ states transformed from ineffective to effective in online collaborative learning. In other words, studies aiming to depict the transformation of online collaborative states and further improve collaboration accordingly remain rare.

Therefore, this study first proposed online collaborative learning guidelines based on CCLT. Then, we revealed the collaborative transformation via learning process data analysis. Furthermore, an optimized mechanism was formulated to provide instructional strategies in line with the transition of collaborative states, and a multi-objective approach was also incorporated to avoid extra cognitive load, as well as maximize the intervention effect. Finally, a quasi-experiment was employed in order to examine the capability of the mechanism in promoting awareness of collaboration and learning achievement.

## 2. Related Work

### 2.1. Cognitive Load in Online Collaborative Learning

Cognitive load is a vital factor related to mental health, which has been assessed by mental load and mental effort [3]. According to Kirschner et al. [5], cognitive load refers to the total working memory needed for learning activities. Working memory involves the processing of new information. When the amount of received new information far exceeds working memory limits, it may cause excessive cognitive load. It was identified that cognitive overload was more prevalent in computer-supported environments due to the complex and diverse interactive situations. In order to scaffold cognitive load improvement in collaborative learning, researchers combined cognitive load theory (CLT) [19] with computer-supported collaborative learning (CSCL) [20] and proposed collaborative cognitive load theory (CCLT) [5]. CCLT presented a general framework for guiding instructors and students on how to conduct effective collaboration from the perspective of cognitive load and contained specific principles, including task complexity, task guidance and support, domain expertise, collaboration skills, team size, team roles, team composition, prior task experience, and prior team experience. CCLT defined the collaborative learning context as the intercourse among the learning task, individual learners, and team.

As noted by Jeong et al. [21], there was a special challenge for online collaborative learning that differed from CSCL settings: the difficulty in regulating students’ behavior due to the lack of direct supervision. Therefore, to design the rational optimized mechanism for ameliorating online collaborative learning, appropriate guidelines corresponding to the online learning context are needed. As such, the study first noted the contextual differences between online collaborative learning and CSCL, as well as corresponding guidelines, mainly concerned with two aspects:

- Learning tasks. Compared with CSCL, as online collaboration is hardly ever carried out spontaneously [22], online learning tasks should be posted in advance for collaborative preparation. During the completion of learning tasks, off-topic activities are more likely to occur due to the self-paced learning pattern of the online environment [23]. Furthermore, instructors struggle to offer guidance to online learners one by one in time. Therefore, prompt, continuous, and personalized support is required for better performance in learning tasks [24].
- Learners. As described above, due to the non-spontaneity of online collaboration, specific arrangements and support are required for successful collaboration. Regarding teamwork, the physically isolated feature of online collaborative environments causes the uncertain states of learners [4], which gives rise to inadequate social and emotional involvement [25].

Combining the online collaborative learning context features with the major idea of CCLT, this study put forward the followed online collaborative learning guidelines in the Table 1.

### 2.2. Online Collaborative Improvement

Researchers have attempted to adopt specific scaffoldings to solve the aforementioned problems in online collaborative learning, including providing different formats of learning materials [26] or pre-training procedures [7], designing grouping strategies [27] and intergroup competition mechanisms [28], proposing collaborative support strategies [29], and designing teacher and embedded experts support [2,24]. Though the affordance of these interventions in online collaborative learning has been fully verified, they only worked to a certain extent (e.g., improved the preparation for collaborative learning) or for a certain facet (e.g., improved teachers’ support or collaborative form).

The evolution in the computational technology field offers new possibilities for learning data analysis and intelligent intervention to boost online collaboration. Research has dealt with identifying learners’ collaborative performance through text mining, social network analysis, epistemic network analysis, etc. For instance, Xie et al. [4] made use of text mining and social network analysis to quantify learners’ contribution and engagement during online collaborative learning. In addition, Saqr and López-Pernas [30] designed a method based on graph-based diffusion centralities for quantifying interactions and identifying student roles within the collaboration. Similarly, the identification of regulatory patterns in online collaborative learning was investigated via content analysis and epistemic network analysis [25]. Regarding data-driven collaborative support, some literature efforts have focused on carrying out online collaborative learning in intelligent collaboration platforms or tutoring systems [31,32].

Except for basic collaborative support from online platforms, researchers attempted to place more emphasis on learners’ personal needs. A personalized feedback approach was proposed according to learners’ behaviors and emotion classification, based on discussion text analysis via a deep neural network model. Researchers found its significant impact on knowledge construction and emotions [18]. Additionally, Troussas et al. [17] designed a mobile game-based learning application, which incorporated personalized peer recommendation and an adaptive learning advice generator based on learners’ knowledge states. The application was evaluated for assisting higher-education students in advancing their knowledge level.

However, to the best of our knowledge, most studies have analyzed the current states of collaborative learning, while it remains unclear how students change to conduct collaboration effectively. As such, the determination of state transformation for ameliorating collaborative performance warrants further exploration. Moreover, the bulk of the intervention-pertinent literature has ignored one potential outcome that might be caused by rough intervention (unable to intervene at an exact time and level): cognitive overload, which impedes efficient interventions and learning performance [5].

### 2.3. Research Questions

To bridge this research gap, the present study aims to construct an optimized mechanism based on collaborative state transformation for managing cognitive load and improving collaborative performance. Starting from quantifying online collaborative features and ascertaining collaborative states through data analysis, the study proposed an evolutionary model to pinpoint the transformation of online collaborative states. Then, an online collaborative optimized mechanism was designed which encompassed collaborative strategies and optimal intervention rules. Finally, the study examined the pedagogical affordance of online learners’ collaborative state transformation, awareness of collaboration, learning achievement, and cognitive load. The specific research questions were as follows:

RQ1: What are the differences between two classes in online learners’ collaborative state transformation, after introducing the proposed collaborative optimized mechanism?

RQ2: What are the differences between two classes in online learners’ awareness of collaboration, learning achievement and cognitive load, after introducing the proposed collaborative optimized mechanism?

## 3. Materials and Methods

Given the scarcity of related work, this study devised a data-driven online collaborative optimized mechanism in light of system dynamics modeling methods. This section introduces the development of the optimized mechanism, including online collaborative features formalization, collaborative states evolutionary modeling, and optimized mechanism design as follows.

### 3.1. Data-Driven Online Collaborative Features Formalization

According to the online collaborative learning guidelines, knowledge mastery and interactive collaboration are key characteristics of online collaborative learning. As the quantitative expression of these two parts is an important basis for collaborative evolutionary modeling, this study provides definitions based on process data in the online learning platform, as follows.

#### 3.1.1. Collaborative Features

**Figure 1:**
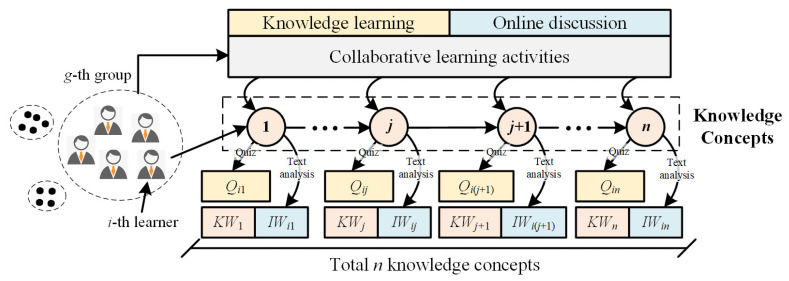
Description of online collaborative learning process.

As the main features of collaborative states, knowledge mastery and effective interaction quantification are necessary for precise state identification [5]. Thus, we have:

- As shown in Figure 1, suppose an online learning course containing total *n* knowledge concepts, the quiz score of i-th learner in j-th knowledge concept (j∈[1,n]) could be represented by $Q_{ij}$($Q_{ij}\in$[0,$\;Q_{j}^{\prime }$]). Then, the knowledge mastery $KM_{ij}$ can be calculated as follows:
  (1) $$KM_{ij}=\frac{Q_{ik}}{Q_{k}^{\prime }},$$
  here we give two thresholds, $\kappa_{1}$ and $\kappa_{2}$ ($\kappa_{1}<\kappa_{2}$), as the boundary values of partial mastery and complete mastery of knowledge, respectively, to determine the levels of knowledge acquisition. If the value is less than $\kappa_{1}$, it indicates that the student has not acquired related knowledge. If the value ranges from $\kappa_{1}$ to $\kappa_{2}$, it denotes the student’ knowledge level as partial mastery; if the value exceeds $\kappa_{2}$, it means the knowledge level is complete mastery.
- The main finding of CCLT indicated that the effective interaction was strongly related to the collaborative theme, which would reduce extraneous information processing and avoid cognitive overload [33]. Online learners generated a large amount of online discussion transcripts, which have been regarded as a sufficient data source for learning analysis [34]. Consequently, we analyzed online discussions to ascertain whether interaction was effective. The study utilized Jieba [35] and GloVe [36] for text segmentation and word embedding and to formalize the keywords of knowledge concepts and interactive textual information as sets $KW_{j}=\left\{kw_{1},kw_{2},\ldots ,kw_{n}\right\}$ and $IW_{ij}=\left\{iw_{i1},iw_{i2},\ldots ,iw_{in}\right\}$, respectively. Then, the effective interaction $EI_{ij}$ could be calculated through analyzing the relatedness of $KW_{j}$ and $IW_{ij}$, which can be expressed as follows:
  (2) $$EI_{ij}=\sum_{k=1}^{n}PC\left(KW_{j},IW_{ij}\right),$$
  where PC() is the similarity calculation function based on Pearson correlation coefficient, which describes the proximity between vectors $KW_{j}$ and $IW_{ij}.$ The higher the value of $EI_{ij}$, the more that theme-related discussions take place. Alternatively, the lower the value of $EI_{ij}$, the less that involved discussions or the more that off-topic discussions take place. After that, a threshold λ was set as the boundary value of the effective interaction level. If $EI_{ij}$ is less than λ, it indicates that the current interaction is not effective enough; and if $EI_{ij}$ is more than λ, it indicates that the current interaction is relatively effective.

#### 3.1.2. Collaborative States

The values of thresholds $\kappa_{1}$, $\kappa_{2}$, and λ given above are determined by historical records and expert knowledge. Accordingly, five collaborative learning states were denoted as follows:

- Initial state *I* (where $KM_{ij}<\kappa_{1},\;EI_{ij}<\lambda )$ implies the preliminary situation that students neither start to acquire knowledge nor carry out effective collaboration.
- Partial mastery of knowledge without effective interaction *M_S_* (where $\kappa_{1}<KM_{ij}<\kappa_{2},\;EI_{ij}<\lambda$) represents learners that have been studying for a while, but have not fully mastered the required knowledge, nor interacted with others effectively.
- Partial mastery of knowledge and effective interaction *M_C_* (where $\kappa_{1}<KM_{ij}<\kappa_{2},\;EI_{ij}>\lambda$) indicates that learners have mastered partial knowledge and conducted effective collaboration with others.
- Complete mastery of knowledge without interaction *L_S_* (where $KM_{ij}>\kappa_{2},\;EI_{ij}<\lambda$) means that the learners have mastered all the knowledge but lack effective collaboration with peers.
- Complete mastery of knowledge and effective interaction *L_C_* (where $KM_{ij}>\kappa_{2},\;EI_{ij}>\lambda$) implies that the learners have almost mastered the needed knowledge entirely and also cooperate with others successfully.

### 3.2. Online Collaborative States Evolutionary Modeling

In this part, we first established a collaborative evolutionary model to describe the transformation of collaborative states in an online course. Within online collaborative learning, the predominantly occurring learning activities could be grouped into three types:

- Basic knowledge acquisition (A1): in the early stage of collaborative learning, learners could acquire basic knowledge through instructions from teachers or utilizing given learning resources.
- Interactive collaboration (A2): under this circumstance, learners are divided into groups for problem-solving or theme discussion regarding a specific issue. In this way, learners not only receive knowledge from others, but can also generate their own knowledge and share it with peers.
- Self-inquiry (A3): in this kind of activity, learners tend to solve problems independently via extra resource searching and utilization.

**Figure 2:**
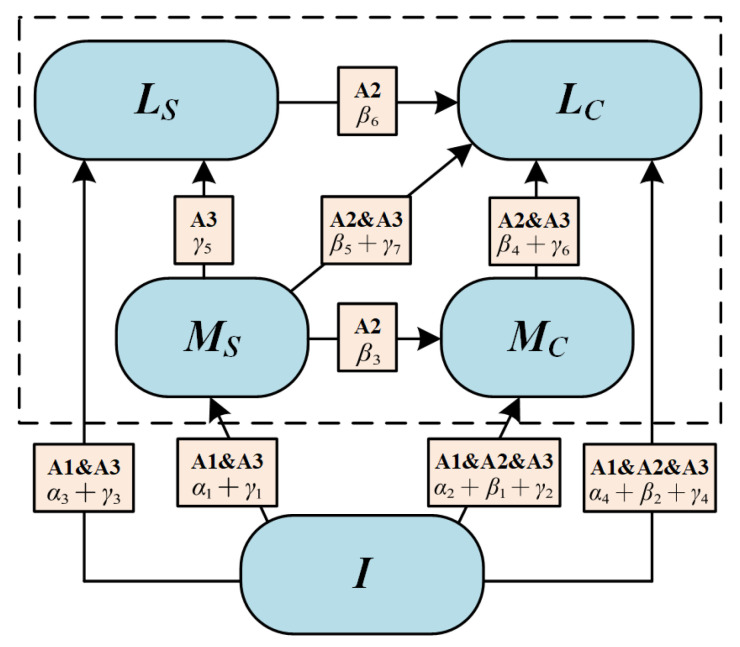
Online collaborative state evolutionary model.

According to the collaborative task setting, we divided learners into G groups, and denoted the group of the *i*-th student as $g_{i}$. Let $CS\in \left\{I^{g_{i}}\left(t\right),\;M_{S}^{g_{i}}\left(t\right),M_{C}^{g_{i}}\left(t\right),L_{S}^{g_{i}}\left(t\right),L_{C}^{g_{i}}\left(t\right)\right\}$ represent the proportion of $g_{i}$ in state *I*, *M_S_*, *M_C_*, *L_S_*, *L_C_* at time *t*, and set the positive constants $\left\{\alpha_{1},\ldots ,\alpha_{4}\right\},\;\left\{\;\beta_{1},\ldots ,\beta_{6}\right\},\;\left\{\;\gamma_{1},\ldots ,\gamma_{7}\right\}$ as state-transition probabilities of learning activities A1, A2, A3, respectively. The state transition is shown in Figure 2.

The whole process of collaborative learning started from the initial state (*I*), then gradually transformed to partial (*M_S_* and *M_C_*) and complete mastery of knowledge (*L_S_* and *L_C_*) through various collaborative learning activities. This process can be described as a time-varying problem of dynamic systems modeling; that is, by regarding the proportion of each state as a function with independent variable *t*, the transformation trend among states can be described as the differential representation of the following function:

(3) $$Y\left(CS\right)=\frac{dCS}{dt},$$

here, Y represents the transformation of collaboration states over time. Based on the theory of system dynamics [37], the transformation trend of each collaborative state can be expressed by the sum of state transition probability with other related states, e.g., the transformation trend of initial state can be calculated as follows:

(4) $$Y\left(I^{g_{i}}\left(t\right)\right)=-I^{g_{i}}\left(t\right)\left[\sum_{v=1}^{2}\left(\alpha_{v}^{g_{i}}+\beta_{v}^{g_{i}}+\gamma_{v}^{g_{i}}\right)+\sum_{v=3}^{4}\left(\alpha_{v}^{g_{i}}+\gamma_{v}^{g_{i}}\right)\right].$$

Similarly, the transformation trend of other states in CS can be calculated in the same way.

### 3.3. Online Collaborative Optimized Mechanism

In order to scaffold online collaborative learning, three strategies were designed in view of different states of online learners:

- Advancing knowledge-oriented strategy S1: this strategy aims to enhance the understanding of knowledge via learning diagnosis and resource recommendation for the learners in *M_S_* or *M_C_* state. We set $\varphi_{1}$ and $\varphi_{2}$ as the implementation weights, respectively.
- Encouraging collaboration-oriented strategy S2: this strategy aims to provide reminder prompts for off-topic discussions and encourage knowledge sharing and evaluation for the learners in *M_S_* or *L_S_* state with implementation weights of $\omega_{1}$ and $\omega_{2}$.
- Mixed strategy S3: this strategy combines S1 and S2, which aims to improve learners’ abilities in both knowledge and interactive aspects and sets $\theta_{1}$ as the implementation weight.

**Figure 3:**
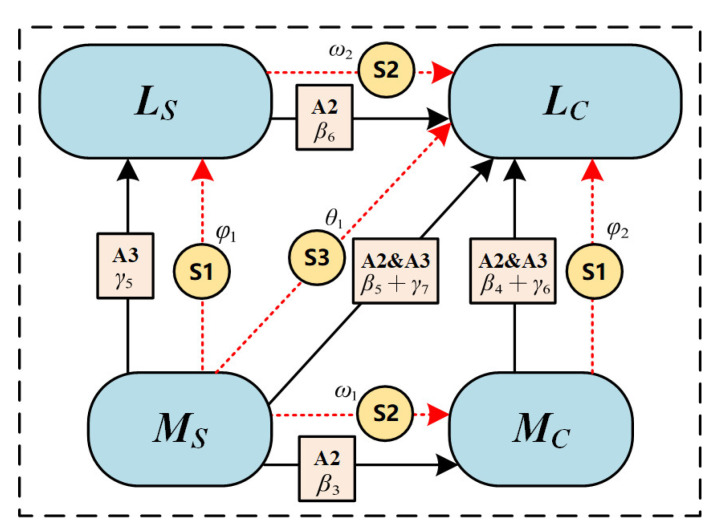
Optimized mechanism implementation in collaborative state evolutionary model.

By integrating the above three strategies into the transformation model of online collaborative learning, we denoted $w_{j}=\left\{\varphi_{1},\varphi_{2},\omega_{1},\omega_{2},\theta_{1}\right\}$ as the set of implementation weight of different strategies; the value of the weights would convey the information of which strategy should be adopted first according to the current state. For example, if $\varphi_{1}$ values highest among other parameters, it indicates that the S1 (advancing knowledge-oriented strategy) is the most needed strategy for the current collaborative state. The corresponding transformation is detailed in Figure 3. Due to the implementation process of the above strategies still belonging to the state-transition process in the evolutionary model, the trend calculation method was similar. Accordingly, for a collaborative state CS, the optimized transformation trend function $Y^{*}()$ can be represented as the sum of Y() and additional strategies influence, as follows:

(5) $$Y^{*}\left(CS\right)=Y\left(CS\right)\pm \sum_{p=1}w_{p}CS_{p},$$

CCLT noted that excessive intervention will increase cognitive load in the process of collaboration, which will interfere with collaborative improvement [33]. Therefore, a multi-objective optimization method was proposed to achieve the optimal collaborative learning performance with lower interference (i.e., less extra load), by further considering the implementation cost of the collaborative optimized mechanism. We denoted m(t) as the number of mechanism implementations at time *t* (*t*∈[0, T]), and C( ) was the cost of cognitive load this mechanism would pay for. Then, the final optimization goal can be expressed as follows:

(6) $$OG\left(t\right)=\underset{m\left(t\right)}{\mathrm{argmax}}\int_{0}^{T}\left[Y^{*}\left(L_{C}^{g_{i}}\left(t\right)\right)-\sum^{\;}C\left(m\left(t\right)\right)\right]dt\;.$$

As online collaborative learning aims to help learners acquire domain knowledge, as well as develop cooperative skills through collaborative learning tasks, which means guiding students to attain the state of *L_C_* as much as possible.

In light of the above optimization objective conditions, the collaborative transformation process can be regarded as the optimal control problem in the dynamic system. Therefore, we employed Pontryagin’s maximum principle [38] and determined the optimal weight, $w_{i}$, as a guidance of the mechanism implementation to improve the overall consequence of collaborative learning with the following steps.

Step 1. Problem description. Considering the limited time and effort of instructors, we investigated how to maximize the probability of learners’ transition to *L_C_* in the dynamic system by choosing the optimal strategy. Hence, it can be described as a traditional optimization task for dynamic programming.

Step 2. Trend expression of each collaboration states. Referring to Equations (4) and (5), the trend of each collaborative state can be calculated by function $Y^{*}()$.

Step 3. Known conditions and objective determination. For each state in CS, the known conditions include: the historical collaborative states of learners, the transition probability between states, and the cognitive load cost of different strategies. The objective is to find the value of implementation probability corresponding to the five strategies, i.e., $\varphi_{1},\varphi_{2},\omega_{1},\omega_{2},\theta_{1}.$

Step 4. Function representation. Based on the previous study [38] and Equation (6), the functions for $\varphi_{1},\varphi_{2},\omega_{1},\omega_{2},\theta_{1}$ can be formalized related to the known conditions above through an equivalence transformation of CS.

Step 5. Iterative solution. Since the small sample size involved, the values of $\varphi_{1},\varphi_{2},\omega_{1},\omega_{2},\theta_{1}$ to achieve the maximum *L_C_* probability can be found by traversing all possible results.

Step 6. Strategy implementation. According to the obtained results of $\varphi_{1},\varphi_{2},\omega_{1},\omega_{2},\theta_{1}$ after every week, the strategy with highest weight will be selected to implement in the next stage.

## 4. Experimental Design

### 4.1. Participants and Study Context

This study employed a quasi-experiment in the spring 2021 semester to evaluate the efficacy of the optimized mechanism. The participants were 91 sophomores, and the average age was 20, with 58 females and 33 males. The imbalance of females and males was in accordance with the university student population. The participants from two intact classes were instructed by the same teacher at a normal university in China, who also took the same online course on a regional online learning platform named ZJOOC (https://www.zjooc.cn/, accessed on 29 June 2021). The selection of the two classes was based on the similarity of their prior knowledge, learning performance, and collaborative competencies. One was randomly assigned as the experimental group (46 students), and the other one was assigned as the control group (45 students). All the participants had never learned the relevant contents before and agreed to participate in the present study.

**Figure 4:**
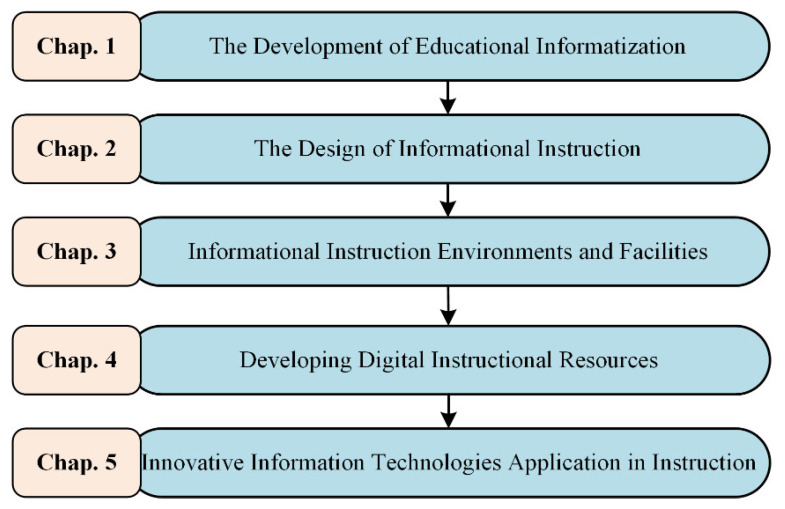
The topics of every chapter of the online course.

The online course pertained to educational technologies theory and application, lasting 10 weeks and involving approximately five chapters of content. Figure 4 presents the topics of every chapter. The majority of online learning activities were watching lecture videos and text materials, collaborating with peers, and taking quizzes (after completing each section) via the online platform. The collaborative learning consisted of theme discussion and problem exploration. As recommended by previous research [39], the study divided students into collaborative groups of 4–6 persons to guarantee a well-balanced group size. The online platform stored numerous log data of the entire learning process, including discourse data, quiz scores, and basic course information, which were available for supporting learners’ collaborative state analysis.

**Figure 5:**
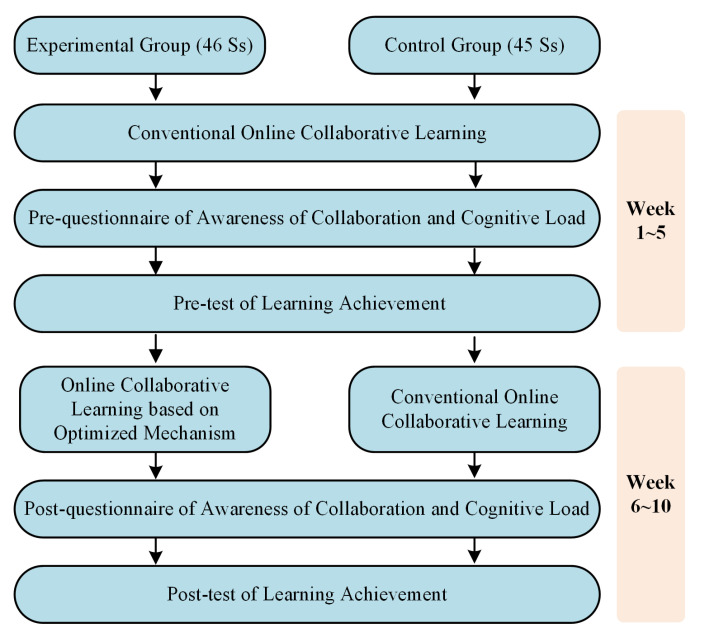
Experimental procedure.

Figure 5 depicts the experimental procedure of the present study. The whole process was structured in two main phases. In the first phase (week 1~week 5), both groups conducted the conventional online collaborative learning. The purpose of this design was threefold: first, this was the pre-training for online collaborative learning, which was required in the aforementioned guidelines (Section 2.1) for adaptation to the online collaborative environments; second, it aimed to accumulate sufficient log data to determine all the parameters in the evolutionary model, in order to shape the optimized mechanism accurately; third, it was designed to avoid learners’ curiosity about online learning influencing the experimental results. Before employing the optimized mechanism, the study first conducted the pre-test of awareness of collaboration, learning achievement, and cognitive load.

In the second phase (week 6~week 10), the experimental group began to learn with the designed optimized mechanism, while the control group still carried out the conventional collaborative learning; that is, the students in the control group did not receive any support strategies. The learning materials and learning tasks were all the same for the two groups.

Specifically for the experimental group, this study acquired the quiz scores of the current section and discussion transcripts at the end of every week. The knowledge mastery, $KM_{ij}$, could be calculated using the Equation (1), and the effectiveness of collaboration, $EI_{ij}$, could be calculated using Equation (2), to ascertain the leaners’ states during online collaborative learning. Through discovering the final optimization goal in Equation (6) based on the cost of cognitive load possibly caused by intervention, we could obtain the values of every weight ($\varphi_{1},\varphi_{2},\omega_{1},\omega_{2},\theta_{1}$). As such, the teacher could know which strategy should be adopted priorly for the optimal improvement effect of online collaboration. At the end of the experiment, this study collected the final scores of the course to evaluate students’ learning achievement and employed the post-test of awareness of collaboration and cognitive load.

### 4.2. Instruments

The transformation of collaborative states, the questionnaire scores of awareness of collaboration and cognitive load, as well as learning achievement, were collected for experimental effect analysis. The transformation of collaborative states was analyzed using the aforementioned model (see Section 3.2) based on learning data recorded on the online platform.

The awareness of collaboration questionnaire was adapted by Lai and Hwang [40]. It consists of three dimensions (trust, communication, and coordination) for a total of five items with a five-point Likert rating; one sample is “When I worked with my group members, I think our conversation was good”. The value of the rating ranges from 5–25. The value of Cronbach’s alpha and Kaiser–Meyer–Olkin (KMO) were 0.73 and 0.79 respectively, and the result of the Bartlett’s test was significant (*p* < 0.05), indicating acceptable reliability and validity. The measurement of cognitive load was developed by Pass and Van Merriënboer [41], consisting of two items (mental effort and mental load) on a nine-point rating scale (1 = minimal effort and very easy; 9 = very hard and very difficult), where the value of the rating ranges from 2–18. The Cronbach’s α value reached 0.83, meaning a high level of reliability. All the questionnaires were reviewed by two researchers who were proficient in related research to ensure the validity.

The pre-test of learning achievement was conducted at the end of the first phase of the experimental procedure, and it consisted of three parts (usual performance, practice work, and mid-term exam). The post-test also consisted of same three parts (usual performance, practice work, and final exam), which was conducted at the end of the second phase. The scores were both given according to the sum of usual performance grade (20%), practice work score (40%), and exam score (40%), with a perfect score of 100. The two exams were developed by two experienced instructors and were evaluated by another experienced instructor to make sure the exams could assess students’ knowledge level of the course.

## 5. Results

### 5.1. Collaborative State Transformation

According to the accumulated learning data, this study identified the values of parameters in the collaborative evolutionary model, as shown in Table 2.

Here the parameters $\kappa_{1},\kappa_{2}$, and λ were determined according to the statistics of historical records. In the preliminary test, the correct answer rate of the quiz for learners without learning experience was 0.23, thus $\kappa_{1}$ = 0.3 was considered to represent partial mastery of knowledge. According to historical records, the correct answer rate reaching 85% always represented a good acquisition of knowledge. Thus $\kappa_{2}$ was set to 0.85 to represent complete mastery of knowledge. As 0.75 was the average value of effective interaction for the historical learners without intervention, 0.75 was taken as the value of λ, indicating the boundary line between ineffective collaboration and effective collaboration.

The parameters $\left\{\alpha_{1},\ldots ,\alpha_{4}\right\},\;\left\{\;\beta_{1},\ldots ,\beta_{6}\right\},\;\left\{\;\gamma_{1},\ldots ,\gamma_{7}\right\}$ were determined based on the collaborative state of 91 learners in the first phase (week 1~week 5). That is, after the first phase of online collaborative learning, the above parameters can be calculated by the average proportion of the corresponding state types in each state. All the values have been reviewed and evaluated by two proficient researchers to guarantee the rationality.

**Figure 6:**
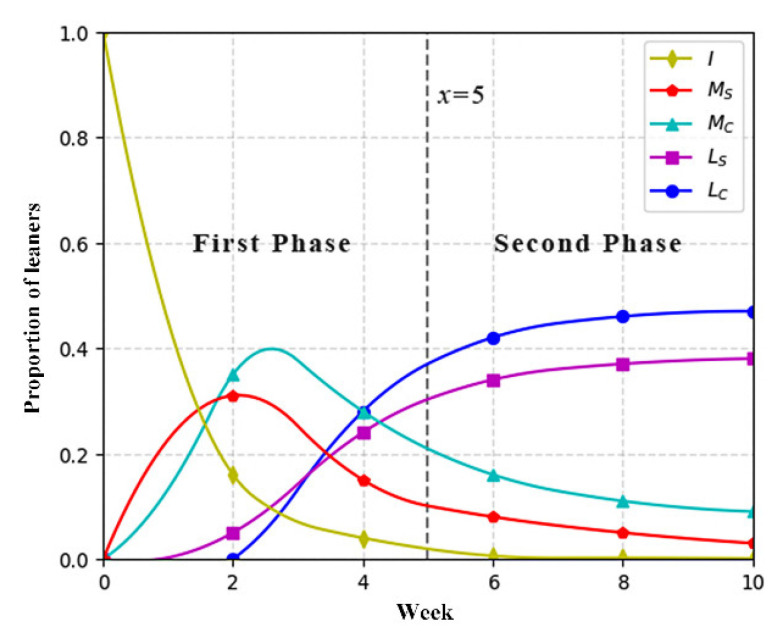
Collaborative state of control group.

**Figure 7:**
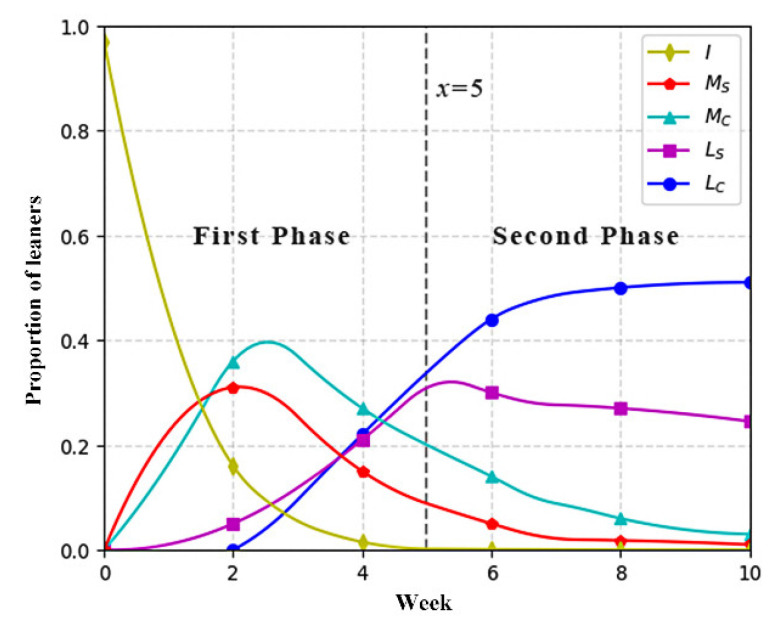
Collaborative state of experimental group.

The study presented the transformation of collaborative states by showing the percentage of each collaborative state in the total (the number of current states/total number). The results are shown in Figure 6 and Figure 7.

The *x*-axis represents the weeks in the whole experiment process, and the *y*-axis represents the proportion of the population in each state to the total number of students. The vertical line in the charts (x = 5) denotes the beginning point of adopting the optimized mechanism, which divided the whole process into two phases.

In the first phase (x ≤ 5), it could be seen that the overall tendency of the collaborative state transformation is quite similar between the two groups from week 1 to week 5, which meant that two groups performed similarly in collaborative learning. In the second phase (x > 5), in view of the chart of the experimental group, it is noticeable that there is an increase in the percentage of students in *L_C_* state, while there is a decline in other states from week 6 to week 10. That indicates that the number of students in *L_C_* state have increased, while the number of students in other state have decreased, indicating that more students advanced their knowledge level and conducted effective interaction after learning with the optimized mechanism. Compared with the chart of the control group, the number of students in *L_C_* state in the experimental group was much higher than that in the control group at the end of the experiment. Additionally, the number of students in the *L_S_* state was much lower than that in the control group. It implies that there was still a part of the students in the control group unwilling to interact with peers or just have shallow interaction. In sum, the results mean that the optimized mechanism did help students to upgrade knowledge level and encourage deeper interactions.

### 5.2. Awareness of Collaboration

Concerning online learners’ awareness of collaboration, the study conducted a one-way ANCOVA, using pre-questionnaire scores as a covariate, optimized mechanism as an independent variable, and the post-questionnaire scores as a dependent variable. The results in Table 3 show that a significant difference existed between the two groups (F = 6.18, *p* < 0.05, η^2^ = 0.066) with a moderate effect size. Additionally, the adjusted mean value and standard deviation errors of the learners’ awareness of collaboration ratings were 18.42 and 0.17 for the experimental group, and 17.82 and 0.17 for the control group. This finding revealed that the online learners in the experimental group displayed better awareness of collaboration than the learners in the control group.

To be specific, the study acquired detail results respecting three dimensions of awareness of collaboration (trust, communication, and coordination) through the one-way ANCOVA. The results implied that there was a significant difference between two groups on communication (F = 17.82, *p* < 0.05, η^2^ = 0.168), while the differences in trust (F = 1.22, *p* = 0.27 > 0.05, η^2^ = 0.014) and coordination were insignificant (F = 0.31, *p* = 0.58 > 0.05, η^2^ = 0.004). In addition, all the adjusted mean values of three dimensions in the experimental group were higher than those in the control group. This implies that students in the experimental group rated much higher in communication and rated slightly higher in trust and coordination.

### 5.3. Learning Achievement

A one-way ANCOVA was conducted to examine the impact of the optimized mechanism on learning achievement by adopting the optimized mechanism as an independent variable, the final score as a dependent variable, and the pre-test scores as a covariate. The results indicated a significant difference (F = 4.59, *p* < 0.05, η^2^ = 0.050) between the two groups (as shown in Table 4). The adjusted mean values of the students’ learning achievement ratings for the experiment and control groups were 83.31 (Std. error = 0.57) and 81.55 (Std. error = 0.58), respectively. Specifically, the subparts (usual performance grade, practical work, and exam score) of learning achievement were analyzed with ANCOVA too. It was found that the optimized mechanism played a positive role in students’ exam performance (F = 4.95, *p* < 0.05, η^2^ = 0.053), while the effects on usual performance grade (F = 0.09, *p* = 0.76 > 0.05, η^2^ = 0.001) and practical work score (F = 3.16, *p* = 0.08 > 0.05, η^2^ = 0.035) were not significant. Accordingly, the optimized mechanism-based collaborative learning was more effective than the conventional collaborative learning with regard to promoting students’ knowledge construction.

### 5.4. Cognitive Load

To analyze the influence of the optimized mechanism on the cognitive load of the students, one-way ANCOVA was utilized by taking the pre-questionnaire scores as a covariate, while the optimized mechanism was the independent variable, and the post- questionnaire scores were a dependent variable. As presented in Table 5, the insignificant effect of the optimized mechanism was found to be F = 0.503 (*p* = 0.48 >0.05, η^2^ = 0.006), indicating that the optimized mechanism did not have a significant effect on learners’ cognitive load in the experimental group. The adjusted mean values and standard deviation errors of the students’ cognitive load ratings were 11.65 and 0.19 for the experimental group and 11.46 and 0.20 for the control group. Consequently, it could be concluded that the optimized mechanism slightly increased the burden of students’ cognition in the experimental group, but it was not heavy compared with the control group.

Concretely speaking, the effects of mental load (F = 1.26, *p* = 0.27 > 0.05, η^2^ = 0.014) and mental effort (F = 0.11, *p* = 0.74 > 0.05, η^2^ = 0.001) were insignificant as well, which also verified the above results. Additionally, the adjusted mean value and standard deviation errors of the students’ mental load ratings were 5.93 and 0.16 for the experimental group and 5.68 and 0.16 for the control group. The values of mental effort in two groups were 5.78 and 0.07, 5.74 and 0.07, respectively. We noticed that the value gap of two groups in mental load was a little larger than that of mental effort. It indicated that students in experimental group experienced slightly more mental load than mental effort.

## 6. Discussion

Combined with CCLT, the present study proposed an online collaborative learning optimized mechanism based on learning data. The mechanism was applied to an online course to examine whether learning with it could ameliorate the transformation of collaborative states, awareness of collaboration, learning achievement, and cognitive load. All the research questions could be fully answered in light of the experiment.

RQ1 analyzed the differences in collaborative state transformation between the experiment and control groups. The results in the first phase (week 1~week 5) showed a similar transformation between the two groups. In other words, this also verified that students of the two groups had a similar level of competency in collaboration and knowledge construction. According to the results in the second phase (week 6~week 10), the percentage of *M_C_*, *M_S_*, and *L_S_* states declined, while the percentage of the ***L_C_*** state increased in the experimental group, revealing that the number of students who performed well in acquiring knowledge and collaboration increased significantly from week 6 to week 10 in the experimental group, indicating that more students began to collaborate effectively after employing the mechanism. Compared with the control group, the findings further verify the conclusion, which is that students in the experimental group outperformed in knowledge acquisition and valid interaction from week 6 to week 10. Overall, it can be concluded that the optimized mechanism was helpful for motivating online learners to acquire knowledge and interact effectively. The finding is compatible with prior work [18,42,43,44], which indicated that personalized feedback and peer assessment enabled online learners to be more involved in collaboration and knowledge building. Considering the special challenge of online collaborative learning [21], we deduce that the results can be attributed to the support that the experimental group received, including learning diagnosis and collaborative guidance, which helped students feel connected with teachers in the isolated online settings, and assisted them in advancing their knowledge level and collaborative engagement.

In terms of RQ2, first, regarding awareness of collaboration, in the light of the experiment results, the students learning with the optimized mechanism performed significantly better in awareness of collaboration. The designed mechanism worked as the catalyst for actively engaging students in teamwork and online discussions. The results conform with the findings of Dao [45], which found that the interaction strategy raised learners’ autonomy of collaboration. The reason for this result is that students in the experimental group would be encouraged to share opinions and evaluate each other. Besides, students who have off-topic discussions would be prompted to concentrate on the learning theme, which leads to more active collaboration. The findings see parallels with the work of Zheng et al. [18] and Chu et al. [46], where they examined the effects of personalized prompts on autonomic and effective collaborative learning. As for the individual dimensions of awareness of collaboration, few studies investigated the specific results. This study found that the proposed optimized mechanism contributed more to students’ “communication” in collaboration, while it impacted little on students’ “trust” and “coordination”. In terms of “communication”, the significant result indicated that the mechanism-encompassed reminder prompts did enhance students’ interaction with each other. In terms of “trust”, similar to a previous study that noted that it was difficult to build and sustain students’ trust in online learning environments [47], our results also reminded us to improve this aspect in future work. As for the “coordination”, one possible reason for the results is that, as the main collaborative activities were theme discussions in our study, the proposed strategies were more suitable for encouraging discussion-based collaboration than project-based collaboration, which required more coordination. Consequently, this would need to be taken into account for further improvement of the mechanism’s generalization to project-based collaboration.

Second, as there were significant differences in learning achievement between the two groups, the results showed that the mechanism played an imperative role in learners’ academic performance. To be specific, “usual performance grade” refers to students’ completion in watching lecture videos and participating in discussions and quizzes, while “practical work score” represents the rating of students’ practical work, including making PowerPoints and micro lesson videos independently. The insignificant results may be due to the fact that both groups were working hard to obtain good basic scores. However, the similar results of two groups in “practical work score” indicated that the proposed optimized mechanism ignored learners’ practical skills development, which should be considered in future improvement plans. The significant result in “exam score” implied a positive contribution from the mechanism in advancing learners’ knowledge construction. It can be deduced that the presented learning diagnosis and reminding prompts for students who lack enough domain knowledge upgraded students’ knowledge in the experimental group, leading to a better performance in the final learning outcomes. The results agree with the finding of Coll et al. [24], which showed that teachers’ feedback in relation to learning content and social participation serves positive functions in terms of knowledge building. This finding is similar to that of the prior study of Karakostas and Demetriadis [29], which also confirms that reminding prompts were beneficial to learning achievement in online collaborative learning.

Finally, regarding cognitive load, the experiment results revealed no significant difference between the experiment and control groups, which indicated that the mechanism did not cause the overloaded cognition of online learners in the experimental group. This finding is in accordance with the suggestions of Oluwajana et al. [11] that certain control of cognitive load is required to help students collaborate effectively. Specifically, the detail results indicated that students in the experimental group underwent similar level of mental load and effort with students in the control group. The finding is different from the work of J.C.Y Sun et al. [48], which found that learners receiving warning feedback would bear significantly increased cognitive load. However, this finding supports the viewpoint of Zheng et al. [18], who revealed that students who received personalized feedback shared a similar feeling of cognitive load with students who received nothing. We argue that it may due to the form of the feedback, as encouragement-based learning prompts, which were also employed in current study, would be more affordable than warning-based learning prompts. An interesting finding was that there was a slightly increased mental load compared with mental effort in the experimental group. This is in line with the main idea of CCLT that increased transactive activities impose loads as learners need to process extra information [5]. As mentioned by a former study, students experience significant cognitive load due to their increased mental load when they receive extra learning feedback [48]. A possible reason for this could be that the extra learning feedback increases the burden of a learner’s mental load, but the optimized mechanism controls it to remain within an affordable range due to the multi-objective optimization method. Therefore, there was no significant difference in mental load between the two groups. Consequently, the multi-objective optimization approach used in the mechanism considered the intervention cost to determine the most-needed strategy for optimal improvement, which worked to prevent students from cognitive capacity overload.

## 7. Conclusions and Future Work

The study aimed to put forward an optimized mechanism, which was concerned with adaptive collaborative learning strategies and the multi-objective optimizing approach, based on collaborative state transformations. The quasi-experiment was used to explore the impact of an optimized mechanism on collaborative state transformation, awareness of collaboration, learning achievement, and cognitive load. The principal findings suggest the potential of the optimized mechanism to facilitate online collaborative learning without adding extra cognitive load.

The findings of this study contain some implications for researchers and practitioners from several related fields. The theoretical value of the present study is to combine collaborative cognitive load theory into an online collaborative learning context, propose the guidelines corresponding to the online context, and devise the optimized mechanism for ameliorating cognitive load and online collaboration. It can offer guidelines to researchers in the fields of online collaborative learning regarding pedagogical design, individualized support, and improvements in considering learners’ cognitive load. Perhaps, it will aid policymakers or website developers in building effective online collaboration communities or platforms. Additionally, this study quantified online learners’ collaborative states accurately based on process data, which do not solely focus on knowledge mastery or interaction situation, but the combination of both facets. The results can support researchers and practitioners who are interested in collaborative state quantification to investigate a more comprehensive collaborative state analysis, taking more student characteristics into account. Moreover, an evolutionary model of collaborative state transformation was depicted, giving a lens to elucidate the conditions that determine effective online collaborative learning transformations for instructors to employ in online instructional scaffolds design. Furthermore, a multi-objective optimization approach was employed in the mechanism to reduce the extra cognitive load caused by collaborative intervention. As indicated by Janssen and Kirschner [33], further exploration and research related to cognitive load improvement is necessary for optimal collaborative application. Therefore, it also provided empirical evidence on cognitive load improvement for related researchers.

However, this study contains several limitations. Considering the heterogeneity in the effect sizes of the various studies, it is still difficult to say in which disciplines or for which school levels the optimized mechanism is particularly suitable from an empirical point of view. Additionally, limited by the corresponding service interface of the experimental platform, the optimized mechanism has not been fully integrated with the online platform to achieve real-time interventions. Therefore, it is our future plan to collect large-scale and heterogeneous learning data, to attain more fine-grained and subject-oriented state analysis, and provide more adaptive learning support through intelligent module development in an open-source or self-developed platform.

## Figures and Tables

**Table 1:** Online collaborative learning guidelines.

Characteristic		Guideline
Learning tasks	Task formats	Choosing adaptive task formats according to learners’ cognitive level to reduce ineffective collaboration.
	Task complexity	Setting up the acceptable task complexity according to instructional objectives and learners’ analysis in advance.
	Task guidance and support	Designing intelligent collaborative support in the online learning platform to provide guidance and avoid off-topic activities.
Learners	Team size	Control the number of the team members to an appropriate size according to the learning task.
	Team roles	Pre-assigning team roles and being able to adjust role arrangement promptly based on practical situations.
	Domain expertise	Acquiring sufficient domain expertise before collaboration to reduce the extraneous cognitive load.
	Collaboration skills	Offering adequate opportunities to familiarize students with online collaboration environments and providing certain support to develop collaboration skills.

**Table 2:** Value of parameters in collaborative evolutionary model.

Parameter	Notes	Values
$\kappa_{1}$, $\kappa_{2}$	Threshold of knowledge acquisition	0.3, 0.85
λ	Threshold of effective collaboration	0.75
$\alpha_{1}$ $\sim \alpha_{4}$	State transition probability of A1	0.3, 0.4, 0.2, 0.1
$\beta_{1}$ $\sim \beta_{6}$	State transition probability of A2	0.2, 0.05, 0.25, 0.3, 0.2, 0
$\gamma_{1}$ $\sim \gamma_{7}$	State transition probability of A3	0.08, 0.05, 0.25, 0.2, 0.2, 0.15, 0.07

Here $\beta_{6}$ = 0 because there are no learners in the existing records who have transformed from *L_S_* to *L_C_*.

**Table 3:** The ANCOVA results for awareness of collaboration.

Group	N	Mean	SD	Adjusted Mean	Std Error	F	η^2^
Experimental group	46	18.65	1.64	18.42	0.17	6.18 *	0.066
Control group	45	17.58	1.97	17.82	0.17		

* *p* < 0.05.

**Table 4:** The ANCOVA results for learning achievement.

Group	N	Mean	SD	Adjusted Mean	Std Error	F	η^2^
Experimental group	46	84.28	6.16	83.31	0.57	4.59 *	0.050
Control group	45	80.56	9.14	81.55	0.58		

* *p* < 0.05.

**Table 5:** The ANCOVA results for cognitive load.

Group	N	Mean	SD	Adjusted Mean	Std Error	F	η^2^
Experimental group	46	11.96	2.39	11.65	0.19	0.503	0.006
Control group	45	11.16	2.61	11.46	0.20		

## Data Availability

The data are not publicly available due to privacy restrictions.
